# Wild Griffon Vultures (*Gyps fulvus*) as a Source of *Salmonella* and *Campylobacter* in Eastern Spain

**DOI:** 10.1371/journal.pone.0094191

**Published:** 2014-04-07

**Authors:** Clara Marin, Maria-Dolores Palomeque, Francisco Marco-Jiménez, Santiago Vega

**Affiliations:** 1 Instituto de Ciencias Biomédicas, Departamento de Producción Animal, Sanidad Animal, Salud Pública Veterinaria y Ciencia y Tecnología de los Alimentos, Facultad de Veterinaria, Universidad CEU-Cardenal Herrera, Alfara del Patriarca, Valencia, Spain; 2 Instituto de Ciencia y Tecnología Animal, Universidad Politécnica de Valencia, Valencia, Spain; Institut National de la Recherche Agronomique, France

## Abstract

The existence of *Campylobacter* and *Salmonella* reservoirs in wildlife is a potential hazard to animal and human health; however, the prevalence of these species is largely unknown. Until now, only a few studies have evaluated the presence of *Campylobacter* and *Salmonella* in wild griffon vultures and based on a small number of birds. The aim of this study was to evaluate the presence of *Campylobacter* and *Salmonella* in wild griffon vultures (n = 97) during the normal ringing programme at the Cinctorres Observatory in Eastern Spain. In addition, the effect of ages of individuals (juveniles, subadult and adult) on the presence were compared. *Campylobacter* was isolated from 1 of 97 (1.0%) griffon vultures and identified as *C. jejuni. Salmonella* was isolated from 51 of 97 (52.6%) griffon vultures. No significant differences were found between the ages of individuals for the presence of *Salmonella*. Serotyping revealed 6 different serovars among two *Salmonella enterica* subspecies; *S. enterica* subsp. *enterica* (n = 49, 96.1%) and *S. enterica* subsp. *salamae* (n = 2, 3.9%). No more than one serovar was isolated per individual. The serovars isolated were *S.* Typhimurium (n = 42, 82.3%), *S.* Rissen (n = 4, 7.8%), *S.* Senftenberg (n = 3, 5.9%) and *S.* 4,12:b[-] (n = 2, 3.9%). Our results imply that wild griffon vultures are a risk factor for *Salmonella* transmission, but do not seem to be a reservoir for *Campylobacter*. We therefore rule out vultures as a risk factor for human campylobacteriosis. Nevertheless, further studies should be undertaken in other countries to confirm these results.

## Introduction

Campylobacteriosis and salmonellosis are the two most prevalent zoonoses worldwide [1]. These zoonoses represent an important public health problem and controlling the disease has become a vital challenge in most countries [1]–[7]. Campylobacteriosis and salmonellosis were responsible respectively for 212,064 and 99,020 cases of illnesses in the EU [1]. Moreover, campylobacteriosis is the most common cause of acute bacterial gastroenteritis in the EU [1], [8], [9]. Apart from acute gastroenteritis, campylobacteriosis may lead to more severe, occasionally long-term sequels such as Guillain-Barré syndrome, reactive arthritis and irritable bowel syndrome [10], [11]. The high and rapidly increasing incidence and the capacity of *Campylobacter* to cause considerable morbidity make campylobacteriosis a public health problem of considerable magnitude [2]. However, compared to *Salmonella*, few outbreaks are reported, and most campylobacteriosis cases are considered to be “sporadic” rather than a part of recognised outbreaks, with a seasonal peak in summer [12].

The existence of *Campylobacter* and *Salmonella* reservoirs in wildlife has been considered a potential hazard to animal and human health; however, the number of wildlife species acting as reservoirs is unknown [13]. *Salmonella* and *Campylobacter* species have been isolated from a wide variety of wild birds, although there is great variation between taxa [14]–[24]. Nevertheless, several studies have suggested that wild birds are important in the epidemiology of human and livestock salmonellosis [25]–[30]. In contrast, other studies suggest that they do not present a major public health hazard, given the low numbers and short duration of salmonella excretion [31], emphasising the controversial nature of the subject matter.

The transmission pathway of *Campylobacter* included direct and indirect contact with infected animals, people and environment [2], [25]–[28]. In this context, the shedding of wild birds faeces into the environment has been identified as a significant reservoir of *Campylobacter* spp. [32]. For example, exposure to contaminated wild bird faeces in playgrounds has been recognised as a potential environmental source of campylobacteriosis, particularly for children [33]. Avian faecal matter found in children's playgrounds was positive for C. jejuni in both dried and fresh samples [34]. Recently, Waldenström et al. [32] reported that to better understand *Campylobacter* biology in wild birds, a number of fundamental questions need to be addressed to elucidate the role of wild birds as reservoirs for *Campylobacter*, to identify host-specific strains, how long wild birds are colonised for and whether the duration and intensity of bacterial shedding vary depending on strain origin. Furthermore, does colonisation of *C. jejuni* have any measurable effects on body condition, or are wild birds asymptomatic carriers similar to chickens?

Specifically, in wild griffon vultures both Molina-Lopez et al. [13] and Millán et al. [15] evaluate the presence of *Salmonella* and, to our best knowledge, only Molina-Lopez et al. [13] evaluate the presence of *Campylobacter*. In this context, Spain holds the most important Griffon vulture population in Europe [35]–[37]. Protection of breeding colonies, limited direct persecution (shooting) and a reduction in the use of poisons have assisted the species' recovery [37]. This would not have been possible without the availability of livestock carcasses, abundant in Spain, and legislation which does not insist on the carcasses being buried or burnt [37].

The aim of this study was to investigate occurrence of *Campylobacter* and *Salmonella* in wild griffon vultures and identify the isolates to species.

## Materials and Methods

The Ethics and Animal Welfare Committee of the Universidad CEU Cardenal Herrera approved this study. All animals were handled according to the principles of animal care published by Spanish Royal Decree 1201/2005 (BOE, 2005; BOE =  Official Spanish State Gazette). The Department of Infrastructure, Planning and Environment of the Valencian Regional Government (Generalitat Valenciana) granted permission to take samples. This study was conducted within the conservation project for endangered species in the Valencia Region.

**Figure 1:**
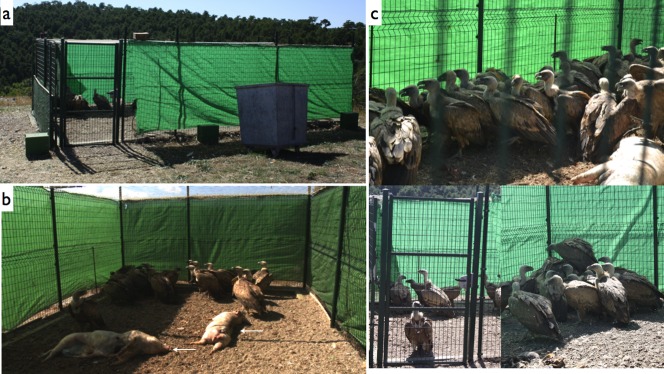
Trap used to capture the wild griffon vultures (*Gyps fulvus*) at Cinctorres Observatory (40°33′N, 0°13′W, Castellón province, Eastern Spain). (a) Cage built *ad hoc* to trap vultures with remote control. (b) Detail of pig carcasses placed on the trap to attract the vultures (white arrows). (c) Details of the large number of vultures analysed.

The study population is located in eastern Spain within the province of Castellón. Wild griffon vultures were captured in September and October 2013 at the Cinctorres Observatory (40°33′N, 0°13′W, Castellón province, Eastern Spain), during the observatory's normal ringing programme. A total of 97 griffon vultures were captured using a remotely activated purpose-built cage (Figure 1). The age of the animals was determined according to the plumage characteristics and the colour of the bill and eye, classified as juvenile (less than 2 years), subadult (between 2 to 5 years) and adult (more than 5 years). From each chick, two cloacal samples were taken using sterile cotton swabs (Cary Blair sterile transport swabs, DELTALAB). We inserted sterile cotton swabs 1 to 2 cm into the cloaca to collect a suitable sample. Each sample was analysed for *Campylobacter* and for *Salmonella* isolation. Additionally, blood samples were collected from the brachial vein (about 5 mL) and transferred to 200 μl pre-refrigerated saline test tubes and stored at 0–4°C. Hematocrit concentration was measured using heparinised micro-hematocrit capillary tubes centrifuged for 5 min at 10,000 rpm and read in a micro hematocrit tube reader.

### Detection of *Campylobacter* spp

Bacteriological culture was performed in accordance with ISO 10272-1∶2006 for the detection of *Campylobacter* spp. (Anonymous, 2006). Moreover, all samples were tested by direct culture. Pre-enriched samples were cultured only if direct culture was negative. Cloacal swabs were directly streaked onto the two selective agar plates (mCCDA and Preston, AES laboratories, Bruz Cedex, France) and incubated at 41.5±1°C for 44±4 hours as reported above. Moreover, cloacal swabs were pre-enriched in 1∶10 vol/vol Bolton Broth (OXOID, Dardilly, France) and then pre-incubated at 37±1°C for 5±1 hours. Then, the pre-enriched broth was incubated at 41.5±1°C for 43±1 hours. Afterwards, 10 μl of the sample was cultured on the two selective agar plates (mCCDA and Preston agar) and incubated as described above. Campylobacter-like colonies were purified on blood agar and identified to species level on the basis of standard procedures comprising tests for hippurate and indoxyl acetate hydrolysis, catalase production, and susceptibility to cephalotin and nalidixic acid.

### Detection of Salmonella spp

The procedure was based on ISO 6579: 2002 recommendations (Annex D). Samples were pre-enriched in 1∶10 vol/vol Buffered Peptone Water 2.5% (BPW, Scharlau, Barcelona, Spain) and then incubated at 37±1°C for 18±2 hours. The pre-enriched samples were transferred onto Semi-Solid Modification Rappaport Vassiliadis agar plate (MSRV, Difco, Valencia, Spain) and incubated at 41.5±1°C for 24–48 hours. The culture obtained in MSRV was inoculated onto Xylose–Lysine–Desoxycholate (XLD, Liofilchem, Valencia, Spain) and Xylose–Lysine–Tergitol-4 (XLT4, Biokar Diagnostics, Pantin Cedex, France) and incubated at 37±1°C for 24–48 hours. After incubation, 5 typical colonies were streaked onto the surface of pre-dried nutrient agar plates (Scharlab, Barcelona, Spain) 37±1°C for 24±3 hours. Then, a biochemical test using API (API-20, bioMerieux, Madrid, Spain) was performed to confirm *Salmonella* spp. Moreover, *Salmonella* strains isolated were serotyped by the Ministry of Agriculture, Fisheries and Food Reference Laboratory (Algete, Madrid, Spain) in accordance with Kauffman-White-Le Minor technique.

### Statistical analyses

We use a generalised linear model (GLM) to compare the body mass and hematocrit against the ages of individuals (Juveniles, subadult and adult). As previously, a GLM which assumed a binomial distribution for *Salmonella* shedding was fitted to the data to determine whether there is a main effect of the ages of individual griffon vultures. A P value of less than 0.05 was considered to indicate a statistically significant difference. Data are presented as least squares means ± standard error of the least squares means. All statistical analyses were carried out using a commercially available software program (SPSS 16.0 software package; SPSS Inc., Chicago, Illinois, USA, 2002).

## Results

All the griffon vultures tested in this study were healthy, according to the hematocrit level and body mass (Table 1). No significant differences were found between ages of individuals for body mass and hematocrit value.

**Table 1:** Measurements of body mass and hematocrit in the Spanish wild griffon vultures (*Gyps fulvus*) according to age.

Age	n	Body mass (g)	Hematocrit (%)
Juveniles	5	8508±76	45.2±0.4
Subadult	11	8250±200	45.8±1.0
Adult	81	7498±297	46.8±1.5
All	97	8447±70	45.4±0.4

The age of the animals was determined according to the plumage characteristics and the colour of the bill and eye (juvenile, less than 2 years; subadult, between 2 to 5 years; adult, more than 5 years). Data are presented as least squares means ± standard error of the least squares means.

n: number of birds analysed.

*Campylobacter* was isolated from 1 of 97 (1.0%) griffon vultures and identified as *C.* jejuni. *Salmonella* was isolated from 51 of 97 (52.6%) griffon vultures. No significant differences were found between the ages of individuals for the presence of *Salmonella* (Table 2).

**Table 2:** Percentage of *Salmonella*-positive wild griffon vultures from different ages.

Age	n	*Salmonella* (%)
Juveniles	5	80.0
Subadult	11	54.5
Adult	81	50.6
All	97	52.6

The age of the animals was determined according to the plumage characteristics and the colour of the bill and eye (juvenile, less than 2 years; subadult, between 2 to 5 years; adult, more than 5 years).

n: number of birds analysed.

Serotyping revealed 6 different serovars among two *Salmonella enterica* subspecies (Table 3); *S. enterica* subsp. *enterica* (n = 49, 96.1%) and *S. enterica* subsp. *salamae* (n = 2, 3.9%). No more than one serovar was isolated per individual. The serovars isolated were *S.* Typhimurium (n = 42, 82.3%), *S.* Rissen (n = 4, 7.8%), *S.* Senftenberg (n = 3, 5.9%) and *S.* 4,12:b[-] (n = 2, 3.9%).

**Table 3:** *Salmonella* serovars isolated from wild griffon vultures (*Gyps fulvus*).

Subspecies	Serovar	n
*enterica*	Monophasic Typhimurium 4,5,12:i: -	40
*enterica*	Monophasic Typhimurium 4,12:i: -	1
*salamae*	4,12:b: -	2
*enterica*	Senftenberg 1,3,19: g,s,t: -	3
*enterica*	Rissen 6,7: f,g: -	4
*enterica*	Typhimurium 4,12:i: 1,2	1

n: number of strains isolated.

## Discussion

Our experiment was carried out to examine colonisation of *Salmonella* and *Campylobacter* in wild griffon vultures. To our knowledge, this is the only study in the scientific literature to evaluate a considerable sample size from a healthy population of wild griffon vultures. Hematocrit values are a useful indicator of the general state of health in raptors [38], [39]. The mean hematocrit values obtained were similar to those described previously in the literature for griffon vultures [40].

The shedding of wild bird faeces into the environment has been identified as a significant reservoir of *Campylobacter* spp. [32] and we tested the hypothesis that wild griffon vultures could also be a potential reservoir for *Campylobacter*. Wild birds may factor into both *Campylobacter* and *Salmonella* epidemiology by coming into contact with farm and food production animals and moving great distances throughout the landscape [41], [42]. Wild birds can function as animal vectors, spreading diseases along migration routes and transferring zoonotic bacteria throughout large parts of the world in faecal droppings [43]. In our study, *C. jejuni* was identified in griffon vulture, in agreement with Molina-Lopez et al. [13] and consistent with observations in other species of wild birds [41], [44]. Recently, Griekspoo et al. [45] demonstrated that *C. jejuni* isolated from wild birds are in general distinct from those isolated from human campylobacteriosis and food animals. Thus, our results suggest that wild griffon vultures are relatively poorly colonised by *Campylobacter* and are likely of little importance in human campylobacteriosis. To verify this, future studies will need to evaluate the genotypes of *Campylobacter* isolates from wild griffon vultures and their relationship to those isolated from human campylobacteriosis.

Nevertheless, the positive percentage of *Salmonella* detected in this study among wild griffon vultures was high (52.6%) and these results are inconsistent with those of other studies, in which the detection was lower than 10% [13], [15], [23]. One possible explanation is due to the low numbers of animals that are members of the same species examined in these previous studies (n = 9, [13]; n = 3, [15]; n = 12, [23]). However, in general, the vast majority of the studies suggest a low presence of *Salmonella* in wild raptors [13], [17], [23], [46], [47]. The most plausible explanation for the high detection rates of *Salmonella* could be associated with the feed. In our study, 82.3% of *Salmonella* isolates belong to *S.* Typhimurium, a serovar of particular public health significance. This is, together with *S.* Enteritidis, one of the most frequently reported serovars involved in human salmonellosis [1], [48]. *Salmonella* Typhimurium has previously been described in griffon vulture [13], [15] and is considered the most common serotype found in wild birds [49]–[51]. In Spain, after the appearance of the first cases of Bovine Spongiform Encephalopathy, griffon vultures almost exclusively fed on swine carcasses [52], [53]. According to the latest studies, *S.* Typhimurium is the most prevalent serotype isolated in swine production [54]–[56]. Although it has previously been posited that raptors may have acquired the bacteria by ingesting small passerines, mice or pigeons [15], [57], our findings suggest the possibility that vultures may be contaminated by ingesting pig carcasses. In raptors, it has been reported that *Salmonella* may indicate infection in other animal populations (e.g. small mammals and passerine birds) [58], [59]. Millán et al. [15] suggest that wild griffon vultures could acquire the bacteria from sources such as livestock carcasses or human residues. However, further studies are necessary to determine the origin of the *Salmonella* reported here and the exact role, if any, of the pig in transmitting the infection to griffon vultures. Nevertheless, our results underline the need to consider the griffon vultures as a potential infection source of this bacterial pathogen, and the zoonotic risk for the general population should be considered. As an example, Davies and Wales [42] recently illustrated the potential of domestic cereal crops to pose a risk of Salmonella exposure for livestock via compound feedstuffs.

All serovars identified have previously been associated with human salmonellosis. Although the other serovars may be considered types rarely associated with human disease, they have also been involved in human salmonellosis. For example, *S.* Rissen, which has previously been described in griffon vulture [13], is commonly isolated from pigs and pork products, whereas it is rarely reported worldwide from human sources, except for Thailand, where in recent years a remarkable rise in infection cases has been emerging [60]–[63]. *S.* Senftenberg has been isolated from wild birds in northern England [16]. Although *S.* Senftenberg is not listed among the top 20 serotypes implicated in human illness [64], the organism is routinely detected in humans and has been recognised in clinical non-human cases of disease (ranked #10 in 2006) and in non-clinical non-human cases (ranked #4), supporting the potential for the emergence of this strain type in human disease [65]. In 2007, the Health Protection Agency Laboratory of Enteric Pathogens reported 51 human isolates of *Salmonella* Senftenberg in England and Wales [66]. In addition, to our best knowledge, *Salmonella* subsp. *salamae* has not been described in wild birds. It has been documented in reptiles and red fox [17], [67] and one case of *S. salamae* infection associated with consumption of reptile meat in humans has been reported [68].

This study showed wild griffon vultures as a risk factor for *Salmonella* infection, but indicates that wild griffon vultures are poor bacterial reservoirs for *Campylobacter* and likely of minimal importance in human campylobacteriosis.
